# Combined Analysis of Transcriptome and Metabolome of Large-Size Triploid Rainbow Trout in Different Seasons

**DOI:** 10.3390/ani16182962

**Published:** 2026-09-20

**Authors:** Zhaonan Li, Changzhong Li, Wenjie Jin, Tianxiu Liang, Shuchen Huang, Jingjing Zhang, Xin Xie, Yanxia Chen

**Affiliations:** 1 Academy of Animal Science and Veterinary, Qinghai University, Xining 810016, China; lizhaonan123456@163.com; 2 Key Laboratory of Plateau Cold-Water Fish Culture and Eco-Environmental Conservation (Co-Construction by Ministry and Province), Ministry of Agriculture and Rural Affairs, Xining 810016, China; 3 School of Ecological and Environmental Engineering, Qinghai University, Xining 810016, China

**Keywords:** triploid rainbow trout, seasonal variation, heat stress, transcriptomics, metabolomics, integrative analysis

## Abstract

Seasonal temperature variation poses a significant physiological challenge for cold-water aquaculture species; however, the molecular mechanisms underlying seasonal adaptation in large-sized triploid rainbow trout (*Oncorhynchus mykiss*) remain largely unexplored. In this study, we integrated liver transcriptomics and LC-MS-based untargeted metabolomics across four seasons (February, May, August, November) to investigate this phenomenon. Seasonal comparisons revealed pronounced molecular reprogramming, with the comparison between May and August showing the largest transcriptomic shift (13,359 differentially expressed genes), while the comparison between May and November exhibited the greatest metabolic divergence (1036 differentially accumulated metabolites). Pathway enrichment analysis identified significant seasonal regulation of oxidative phosphorylation, ribosome, proteasome, and TCA cycle pathways at the transcript level, alongside alterations in amino acid biosynthesis, fatty acid biosynthesis, arachidonic acid metabolism, and linoleic acid metabolism at the metabolomic level. Integrated network analysis further elucidated key gene–metabolite correlations. Integrated network analysis identified season-specific regulatory hubs: Ferroptosis (L-cystine/L-cysteine correlations) dominated the LLF-to-LLM transition; the TCA cycle (citric, fumaric, malic, succinic, and α-ketoglutaric acids) was most responsive from LLF to LLA; Arachidonic acid metabolism linked prostaglandins/thromboxane B2 with PC-class phospholipids from LLM to LLN; and the pentose phosphate pathway (*rbks*, *g6pd*, and *rpe* positively correlated with ribose-1-phosphate) characterized the LLA-to-LLN transition. These findings suggest that large-sized triploid rainbow trout mount a coordinated adaptive response to seasonal temperature fluctuations: during warm months (August), enhanced TCA cycle activity and oxidative phosphorylation meet increased energy demands, accompanied by the accumulation of protective antioxidants such as carnosine and spermine; during cold months (February and November), protein synthesis and ribosomal pathways predominate. Collectively, this study elucidates the molecular architecture of seasonal adaptation in triploid rainbow trout and provides a foundation for enhancing stress resilience and optimizing aquaculture management.

## Simple Summary

Seasonal temperature fluctuations pose a major challenge for cold-water aquaculture species such as rainbow trout. This study investigated hepatic transcriptomic and metabolic responses of large triploid rainbow trout across four seasons (February, May, August, and November). In August, fish upregulated energy metabolism pathways and synthesized protective compounds, potentially reflecting physiological adjustments associated with seasonal temperature, whereas in February and November, gene expression shifted toward protein maintenance and core survival functions. Lipid metabolism underwent pronounced remodeling in November. These findings clarify the molecular basis of seasonal acclimatization in triploid rainbow trout and provide a reference for optimizing management strategies during the physiologically vulnerable summer period, supporting more sustainable aquaculture practices.

## 1. Introduction

Rainbow trout (*Oncorhynchus mykiss*), a member of the Salmonidae family, holds significant ecological value worldwide and is the most extensively farmed carnivorous fish species [1]. Its strong adaptability to diverse aquatic environments, rapid growth rate, and high market demand have established it as a cornerstone of global aquaculture. The triploid rainbow trout, an all-female variant produced by crossing diploid and tetraploid individuals, possesses three sets of chromosomes, rendering it incapable of meiosis and thus unable to produce gametes. The absence of gonadal development results in accelerated growth, superior flesh quality, larger body size, and higher aquaculture yields, making it highly valuable for commercial production [2]. Current research on triploid rainbow trout has primarily focused on energy metabolism [3,4], developmental processes [5,6], and gene expression at different growth stages [7,8]. However, a systematic understanding of the physiological and metabolic regulation of large-sized triploid rainbow trout under varying seasonal environments remains limited. In particular, temperature fluctuations, a key environmental factor, may induce unique metabolic regulatory mechanisms, influencing energy allocation, stress responses, and overall physiological performance. Investigating these adaptive processes is crucial for optimizing aquaculture strategies and ensuring the sustainable production of triploid rainbow trout under changing environmental conditions.

Aquaculture is intricately linked to its surrounding environment, rendering it highly susceptible to the impacts of climate change. Rising global temperatures have led to increased water temperatures, which directly or indirectly influence the growth, metabolism, and survival of numerous aquaculture species. Among the various environmental factors, temperature serves as a fundamental abiotic driver of growth and survival in aquatic organisms, playing a pivotal role in the sustainability of aquaculture [9]. During colder seasons, declining water temperatures typically slow metabolic rates in fish, prompting a shift in energy allocation toward essential life-sustaining processes. This physiological adjustment alters lipid deposition patterns, leading to variations in muscle fat content that, in turn, affect growth efficiency and meat quality [10]. Conversely, during warmer months, elevated water temperatures stimulate metabolic activity, accelerate oxidation processes, and increase respiration rates. This heightened metabolism leads to increased oxygen consumption, ammonia excretion, and carbon dioxide release, further exacerbating the decline in dissolved oxygen levels. Such physiological changes can disrupt respiratory metabolism, induce stress responses, and elevate disease susceptibility in farmed fish [11]. Rainbow trout, a cold-water species that is highly sensitive to temperature fluctuations, exhibits distinct physiological and metabolic adaptations influenced by seasonal variations in temperature and dissolved oxygen, as well as by body size and age [12]. Research has identified an optimal temperature range of 10 to 18 °C for rainbow trout, beyond which physiological stress responses are triggered, potentially compromising metabolic balance and overall health. Both temperature and dissolved oxygen levels play critical roles in determining growth performance and physiological well-being, with their interaction exerting a profound influence on metabolic regulation and aquaculture productivity [13].

Although seasonal temperature fluctuations are known to affect the physiology of farmed salmonids, the molecular mechanisms underlying the coordinated regulation of gene expression and metabolic pathways during seasonal acclimatization in large-sized triploid rainbow trout remain poorly understood. Previous studies have largely relied on single-omics approaches, which are insufficient to capture the integrated molecular responses underlying seasonal physiological regulation. To address this gap, the present study employed an integrative transcriptomic and metabolomic approach to systematically characterize the dynamic regulation of gene expression and metabolic profiles in large-sized triploid rainbow trout across different seasons. By providing deeper molecular insights into the seasonal adaptation mechanisms of this cold-water economic fish species, our findings advance the understanding of its physiological responses and establish a scientific basis for future breeding improvement and research on environmental adaptability in cold-water aquaculture.

## 2. Materials and Methods

### 2.1. Sample Collection

Based on the study conducted by Meng Yuqiong et al. [14], which measured the annual water temperature in the Longyangxia Reservoir, the lowest temperature was recorded in February, while the highest occurred in August. June and September are relatively suitable for the growth of triploid rainbow trout. Accordingly, large-sized triploid rainbow trout (2 ages) were collected from the Longyangxia Reservoir, located in Minze, Qinghai Province. Triploid rainbow trout were identified using flow cytometry (CytoFLEX, Beckman Coulter, Brea, CA, USA), and all experimental fish were all female stock. Fish were sampled at the end of February (designated LLF: large-sized triploid rainbow trout collected in late February; mean water temperature: 4.7 °C; mean body mass: 2.81 ± 0.22 kg), May (LLM: large-sized triploid rainbow trout collected in late May, 10.3 °C; 2.96 ± 0.28 kg), August (LLA: large-sized triploid rainbow trout collected in late August; 20.5 °C; 3.01 ± 0.13 kg), and November (LLN: large-sized triploid rainbow trout collected in late November; 11.4 °C; 3.21 ± 0.35 kg), with the values in parentheses corresponding to the mean water temperature recorded at each sampling time point. Water temperature was the only environmental parameter recorded; other parameters were not monitored concurrently. Our sampling targeted seasonal temperature extremes and transitional phases (pre- and post-growth season), rather than the mid-growing season. We therefore selected May and November as the sampling time points for the transitional phases. Six fish were sampled at each time point, and all were anesthetized using MS-222 (Tricaine methanesulfonate, Sigma-Aldrich, St. Louis, MO, USA) 1:10,000). Fish were fasted for 24 h prior to sampling to minimize the confounding effect of feeding status on hepatic transcriptomic and metabolomic profiles. After dissection, liver tissues were collected and immediately frozen in liquid nitrogen. The samples were then transported to the laboratory and stored at −80 °C for subsequent transcriptome and metabolome analyses.

### 2.2. Transcriptome Profiling

Total RNA was isolated from each sample using TRIzol Reagent (Thermofisher, Waltham, MA, USA) [15]. RNA concentration and purity were assessed using a Nanodrop 2000 spectrophotometer by measuring the OD260/280 ratio (Thermo Fisher Scientific, Waltham, MA, USA). The integrity of RNA was verified with the Agilent 2100 (Agilent Technologies, Santa Clara, CA, USA), ensuring a RIN value greater than 7. cDNA libraries were constructed using Solarbio (RP1105) (Beijing Solarbio Science & Technology Co., Ltd., Beijing, China). Only libraries that met the expected standards were sequenced using the Illumina HiSeq 2500 platform (Illumina, San Diego, CA, USA) [16]. Raw data were filtered with Cutadapt (1.9.1) [17] to remove reads containing adapters, those with a base content exceeding 5%, and low-quality reads, resulting in high-quality clean data. Subsequently, HISAT2 (2.0.5) and HTSeq (0.9.1) [18] were employed to map clean reads against the rainbow trout reference genome (*Oncorhynchus mykiss* genome assembly USDA_OmykA_1.1—NCBI—NLM, reference genome GCF_013265735.2). Overlapping fragments were combined into components, and transcript sequences within each component were identified using the De Bruijn graph approach [19]. DESeq2 (V1.26.0) [20] was utilized to identify differentially expressed genes (DEGs), with criteria set at |log_2_(FC)| ≥ 2 and FDR < 0.05. Gene Ontology (GO) and Kyoto Encyclopedia of Genes and Genomes (KEGG) functional annotations were performed using R software (4.2.1), with a significance threshold of *p* < 0.05 for enriched GO terms or signaling pathways. The raw transcriptome sequencing data have been deposited in the NCBI Sequence Read Archive (SRA) and are accessible under BioProject accession number PRJNA1295884 (https://dataview.ncbi.nlm.nih.gov/object/PRJNA1295884?reviewer=q8u8fe21gaocldhpf9vqma93tl, accessed on 17 September 2026). To validate the transcriptome-sequencing results, total RNA was extracted from liver tissues using an RNA extraction kit (TIANGEN, Beijing, China, DP419). RNA concentration, purity and integrity were assessed by agarose gel electrophoresis, a NanoDrop 2000 spectrophotometer and an Agilent 2100 Bioanalyzer system. Samples with an RNA integrity number (RIN) > 7 were retained for subsequent experiments. RNA was reverse-transcribed into cDNA using a reverse-transcription kit (TIANGEN, KR106). Eight differentially expressed genes (DEGs) with specific expression patterns were selected for qPCR validation. Gene-specific primers were designed with Primer Premier 5 software and synthesized by Sangon Biotech (Shanghai, China); detailed primer sequences are listed in Table 1. β-actin (*actb*) was used as the reference gene, and the relative gene expression levels in liver tissues were calculated using the 2^−ΔΔCt^ method, and the corresponding log_2_FC values were further derived for transcriptome-qPCR correlation comparison. Candidate genes for validation were selected according to the following criteria: (i) statistically significant and directionally consistent differential expression across multiple pairwise seasonal comparisons, defined by a fold-change threshold of |log_2_FC| ≥ 2 and a false discovery rate of FDR < 0.05; (ii) inclusion of both up-regulated (*suclg1*, *asns*, *bdh1*, and *olah*) and down-regulated (*rpp14*, *acsl4a*, *adpgk*, and *sdsl*) genes to ensure balanced representation of bidirectional transcriptional responses; and (iii) functional association with the core metabolic pathways central to this study—namely, the tricarboxylic acid (TCA) cycle, lipid metabolism, and amino acid metabolism—thereby providing a functionally representative cross-validation of the transcriptomic dataset.

### 2.3. Metabolomic Profiling

Liver tissue samples were homogenized, followed by centrifugation to remove cellular debris. Metabolites were extracted using solid-phase extraction. An untargeted metabolomics study was conducted utilizing liquid chromatography-mass spectrometry (LC-MS) [21]. *LC-MS acquisition parameters were as follows: chromatographic separation was performed on an ACQUITY UPLC HSS T3 column (2.1 mm × 100 mm, 1.8 μm, Waters, Milford, MA, USA) at 40 °C with a flow rate of 0.3 mL/min. Mobile phase A was 0.1% formic acid in water (*v*/*v*), and mobile phase B was 0.1% formic acid in acetonitrile (*v*/*v*). The gradient elution program was set as follows: 0 min, 5% B; 0–2 min, 5–20% B; 2–8 min, 20–80% B; 8–11 min, 80–100% B; 11–13 min, 100% B; 13.0–13.1 min, 100–5% B; 13.1–15 min, 5% B. The injection volume was 5 μL, and electrospray ionization (ESI) was operated in both positive and negative modes. The raw mass spectrometry data files (.raw) were imported into Compound Discoverer 3 [22] for spectral processing and database matching, yielding qualitative and quantitative metabolite data. Rigorous quality control measures were implemented to ensure data accuracy and reliability. QC pooled samples were repeatedly injected throughout the analytical sequence, and metabolites with relative standard deviation (RSD) > 30% in QC samples were excluded for subsequent analysis. The metabolomics data were subsequently processed using the metaX 1.0.3 [23] software package. Identified metabolites were annotated utilizing the KEGG database (https://www.genome.jp/kegg/pathway.html, accessed on 15 September 2026), the HMDB database (https://hmdb.ca/metabolites, accessed on 15 September 2026), and the LIPIDMaps database (http://www.lipidmaps.org/, accessed on 15 September 2026). Principal component analysis (PCA) and partial least squares discriminant analysis (PLS-DA) were performed to elucidate metabolic differences among sample groups after data normalization and scaling. A permutation test with 200 permutations was carried out for the PLS-DA model to exclude model over-fitting. Differentially abundant metabolites (DAMs) were identified based on the criteria of variable importance in projection (VIP) > 1, *p*-value < 0.05, and fold change (FC) ≥ 2 or FC ≤ 0.5 [24]. Finally, to elucidate the biological significance of the identified metabolites, metabolic pathway analysis was conducted. The metabolomics dataset generated during the current study is available in the MetaboLights repository (https://www.ebi.ac.uk/metabolights/, accessed on 15 September 2026) under the accession ID MTBLS15014. The complete data can be accessed at https://www.ebi.ac.uk/metabolights/MTBLS15014, accessed on 15 September 2026.

### 2.4. Integration of Transcriptomics and Metabolomics Data

To thoroughly investigate the molecular response of triploid rainbow trout to seasonal variations, we conducted an integrated analysis of transcriptomic and metabolomic data. For the co-pathway analysis, differentially expressed genes (DEGs) and differentially accumulated metabolites (DAMs) were mapped to the Kyoto Encyclopedia of Genes and Genomes (KEGG) pathways. Pathways demonstrating significant enrichment (*p* < 0.05) in both omics datasets, along with pathways associated with heat stress, were identified as key participants in the response. Subsequently, the Pearson rank correlation coefficient was calculated to evaluate the relationships between gene and metabolite expression levels, and a correlation heatmap was generated for visualization.

## 3. Results

### 3.1. Transcriptome Analysis

#### 3.1.1. Transcriptome Sequencing Quality

RNA sequencing was conducted on liver samples from large-sized triploid rainbow trout. Prior to sequencing, all 24 RNA samples passed quality control, with RIN values greater than 7 and OD260/280 ratios ranging from 1.8 to 2.0. As summarized in Table 2, clean reads constituted over 94% of the raw reads across all samples. The GC content ranged from 46.38% to 50.87% across the 24 libraries, with Q20 values exceeding 97% and Q30 values surpassing 94% for each sample. Collectively, these metrics indicate that the transcriptome sequencing data are of high quality and are suitable for subsequent differential expression and pathway enrichment analyses.

**Figure 1:**
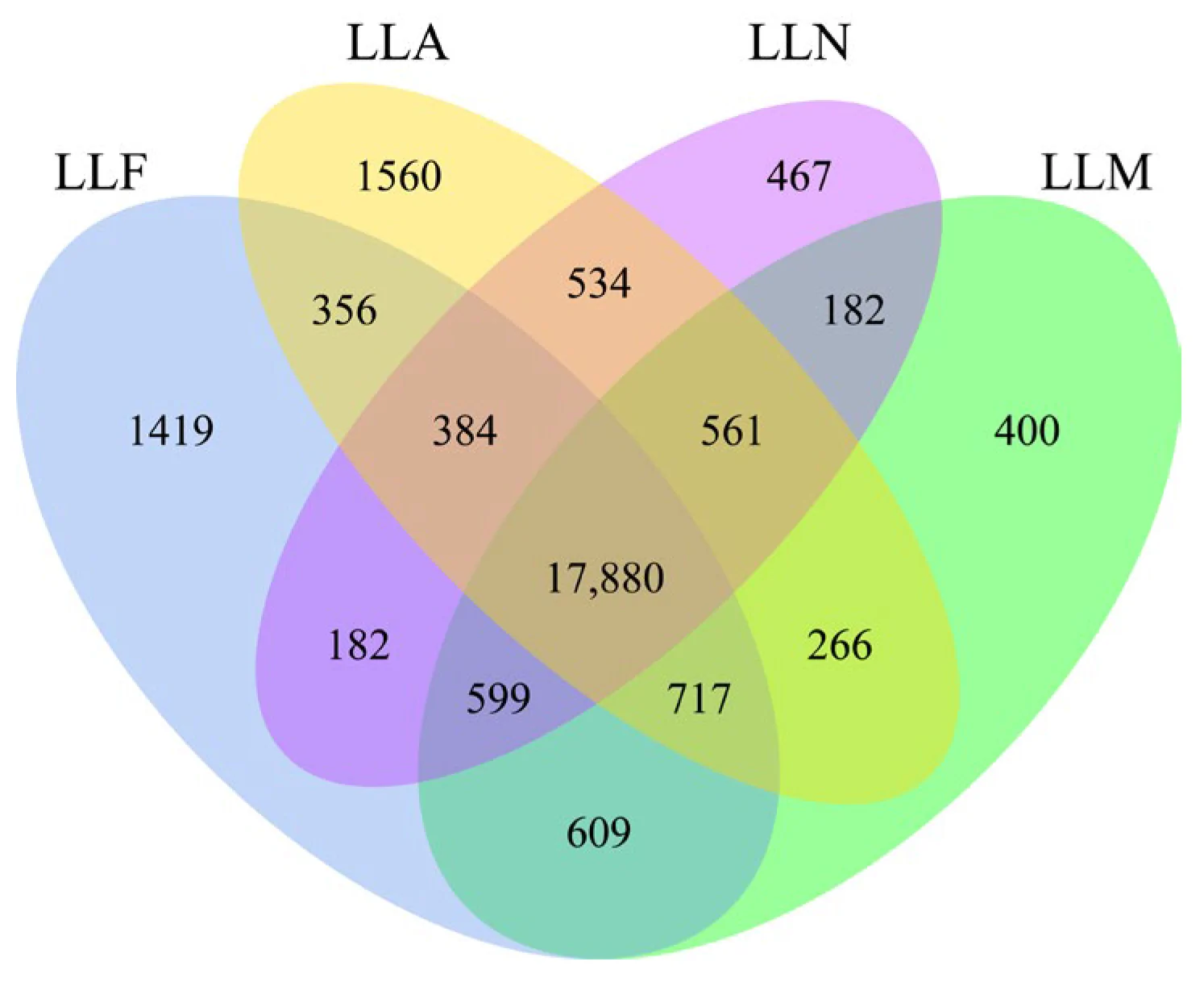
Venn diagram of co-expressed genes.

A total of 1419, 400, 1560, and 467 uniquely expressed genes were identified in the LLF, LLM, LLA, and LLN groups, respectively. Pairwise comparisons revealed that the fewest shared genes occurred between the LLF and LLN groups, with a total of 182 common genes identified in both the LLF vs. LLN and LLM vs. LLN comparisons. In contrast, the comparison between LLA and LLN showed the greatest overlap, with a total of 534 shared genes. Notably, 17,880 genes were commonly expressed across all four groups (Figure 1).

#### 3.1.2. Analysis of Differentially Expressed Genes

**Figure 2:**
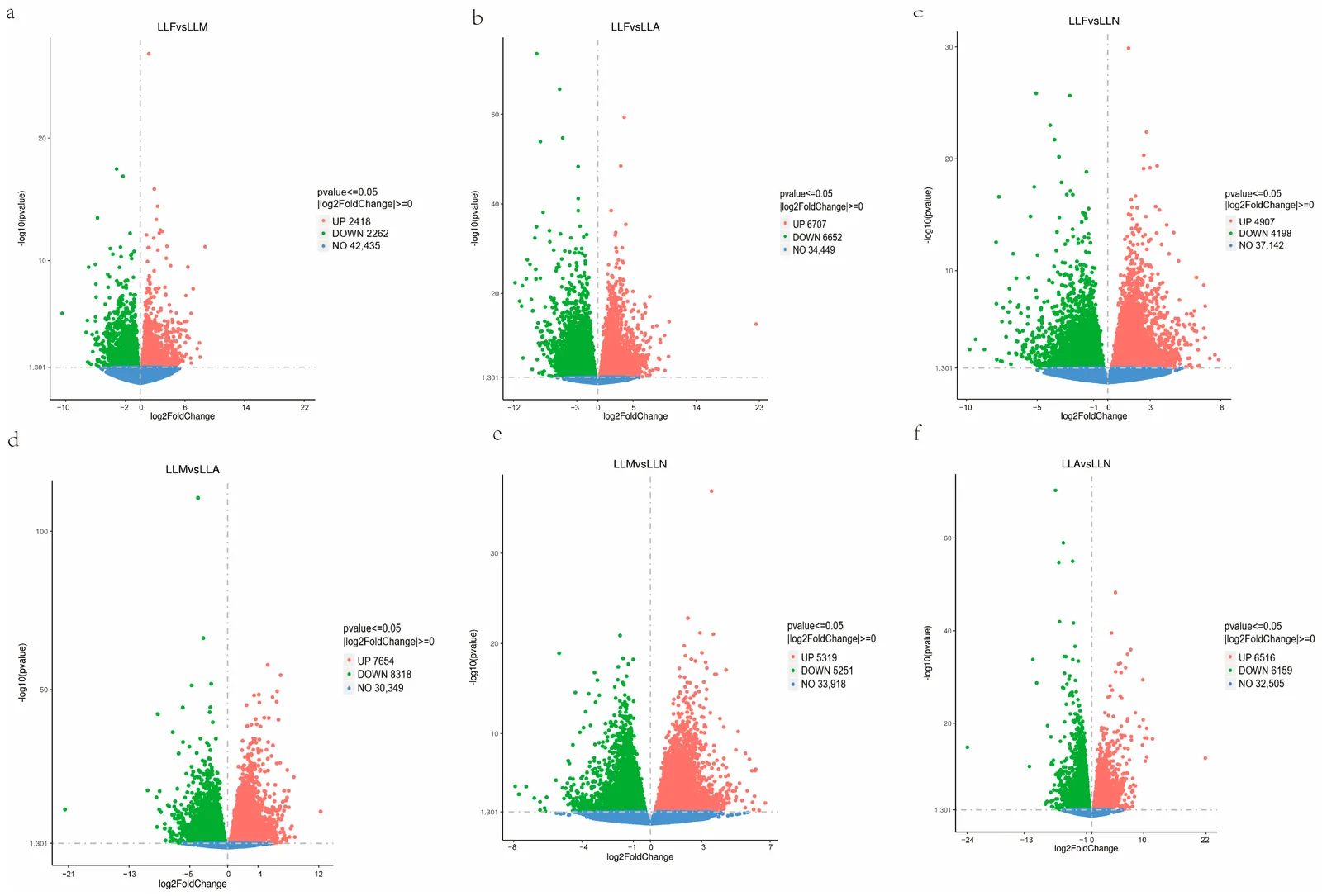
Volcano map of differentially expressed genes. Notes: (**a**) LLF vs. LLM; (**b**) LLF vs. LLA; (**c**) LLF vs. LLN; (**d**) LLM vs. LLA; (**e**) LLM vs. LLN; (**f**) LLA vs. LLN. Each dot represents an individual gene. Genes significantly upregulated and downregulated are marked in red and green, respectively, while non-significant genes are shown in blue. The thresholds for significant differential expression were set at |log_2_FC| ≥ 2 and FDR < 0.05. Vertical dashed lines represent |log_2_FC| = 2, and the horizontal dashed line represents FDR = 0.05 (−log_10_(FDR)) = 1.301.

To investigate the differences in gene expression between triploid rainbow trout in different seasons, a volcano plot analysis was performed. The LLM vs. LLA comparison group showed the largest number of differentially expressed genes (DEGs), with 15,972 DEGs, while the LLF vs. LLM comparison group exhibited the fewest DEGs, with 4680. Except for the comparison groups between May and August, where the number of downregulated genes exceeded the number of upregulated genes, all other comparison groups showed the opposite trend (Figure 2). In the LLF vs. LLM group, a total of 4680 DEGs were identified, with 2418 upregulated and 2262 downregulated. The LLF vs. LLA group yielded 13,359 DEGs, with 6707 upregulated and 6652 downregulated. The LLF vs. LLN group identified 9105 DEGs, with 4907 upregulated and 4198 downregulated. The LLM vs. LLA group showed 15,972 DEGs, with 7654 upregulated and 8318 downregulated. In the LLM vs. LLN group, 10,570 DEGs were detected, with 5319 upregulated and 5251 downregulated. Finally, the LLA vs. LLN group identified 12,675 DEGs, with 6516 upregulated and 6159 downregulated.

#### 3.1.3. GO Enrichment Analysis of Differentially Expressed Genes

**Figure 3:**
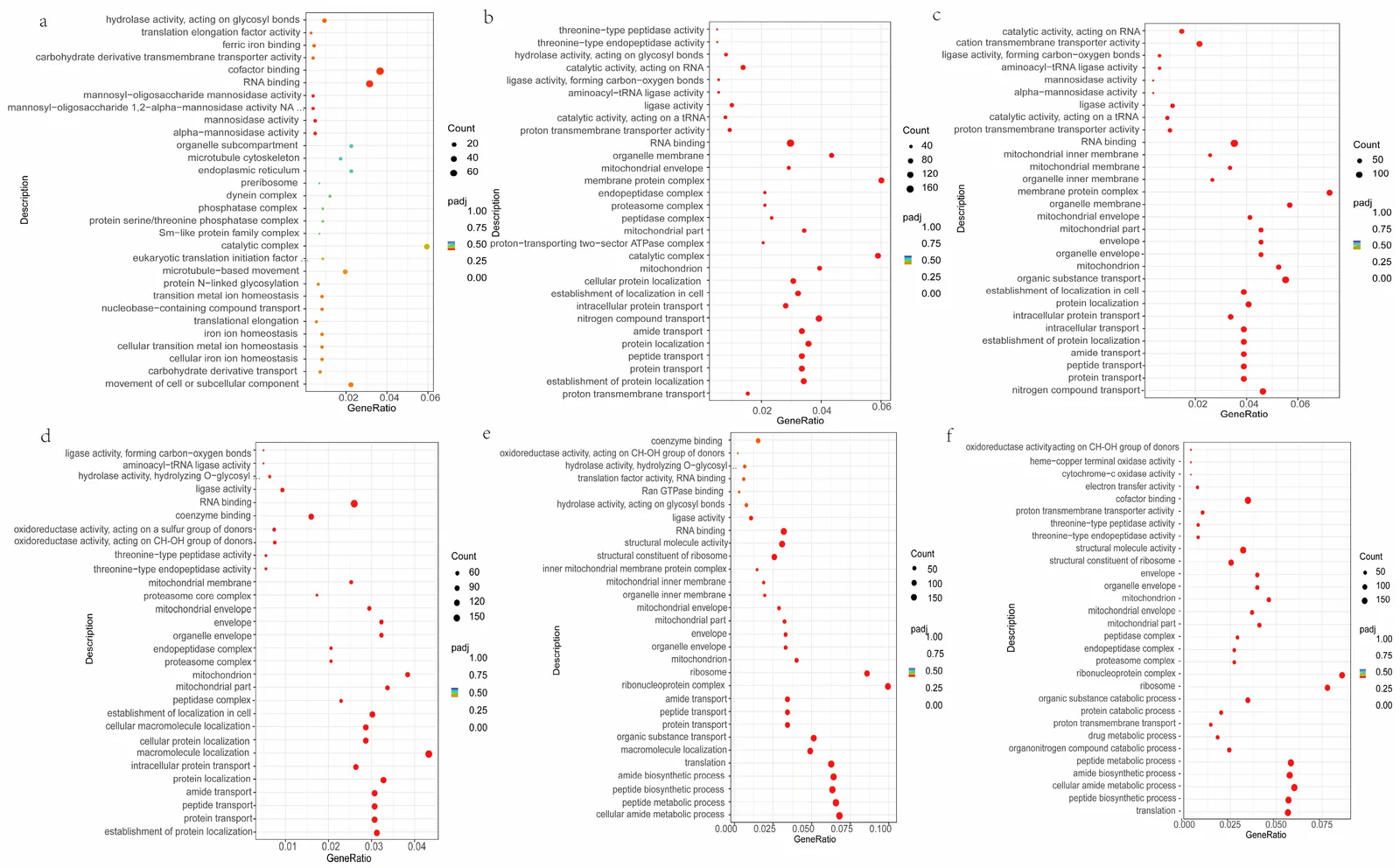
GO enrichment analysis bubble chart. Notes: (**a**) LLF vs. LLM; (**b**) LLF vs. LLA; (**c**) LLF vs. LLN; (**d**) LLM vs. LLA; (**e**) LLM vs. LLN; (**f**) LLA vs. LLN. Bubble size represents the number of enriched genes, and bubble color corresponds to adjusted *p*-value (padj).

In the comparison between the LLF and LLM groups (Figure 3a), the three most significantly enriched Gene Ontology (GO) terms were all associated with mannosidase activity. Notably, the majority of annotated genes were upregulated, with 9 out of 10 genes linked to alpha-mannosidase activity, indicating enhanced glycosylation-associated processes. In the LLF versus LLA group comparison (Figure 3b), enriched terms included proton transport and protein localization, where most genes were also upregulated, with 48 out of 49 genes associated with proton transport, suggesting active cellular trafficking. In the LLF versus LLN group comparison (Figure 3c), terms related to RNA binding and mitochondrial or organelle envelope were enriched; however, downregulated genes predominated in these categories, as evidenced by 45 out of 61 genes associated with the mitochondrion, pointing to reduced RNA metabolism and organelle function. In the comparison between the LLM and LLA groups (Figure 3d), enriched GO terms were primarily related to ligase activity, mitochondrial components, and proteasome complexes, with the majority of annotated genes being downregulated, exemplified by 150 out of 150 genes associated with mitochondrial terms, indicating reduced organelle function and protein turnover. Among the comparisons involving LLN, the most pronounced patterns were observed in the LLM versus LLN group (Figure 3e) and the LLA versus LLN group (Figure 3f). In these contrasts, ribosome- and ribonucleoprotein-related terms were highly enriched but predominantly downregulated, with 133 out of 137 downregulated genes for the ribosome term in the LLA versus LLN group. This widespread downregulation of translation-related machinery suggests a marked reduction in protein synthesis capacity during autumn, irrespective of the preceding seasonal comparison. Collectively, these results reveal season-specific functional reprogramming, with a particularly strong suppression of ribosomal activity in the LLN group.

#### 3.1.4. KEGG Enrichment Analysis of Differentially Expressed Genes

**Figure 4:**
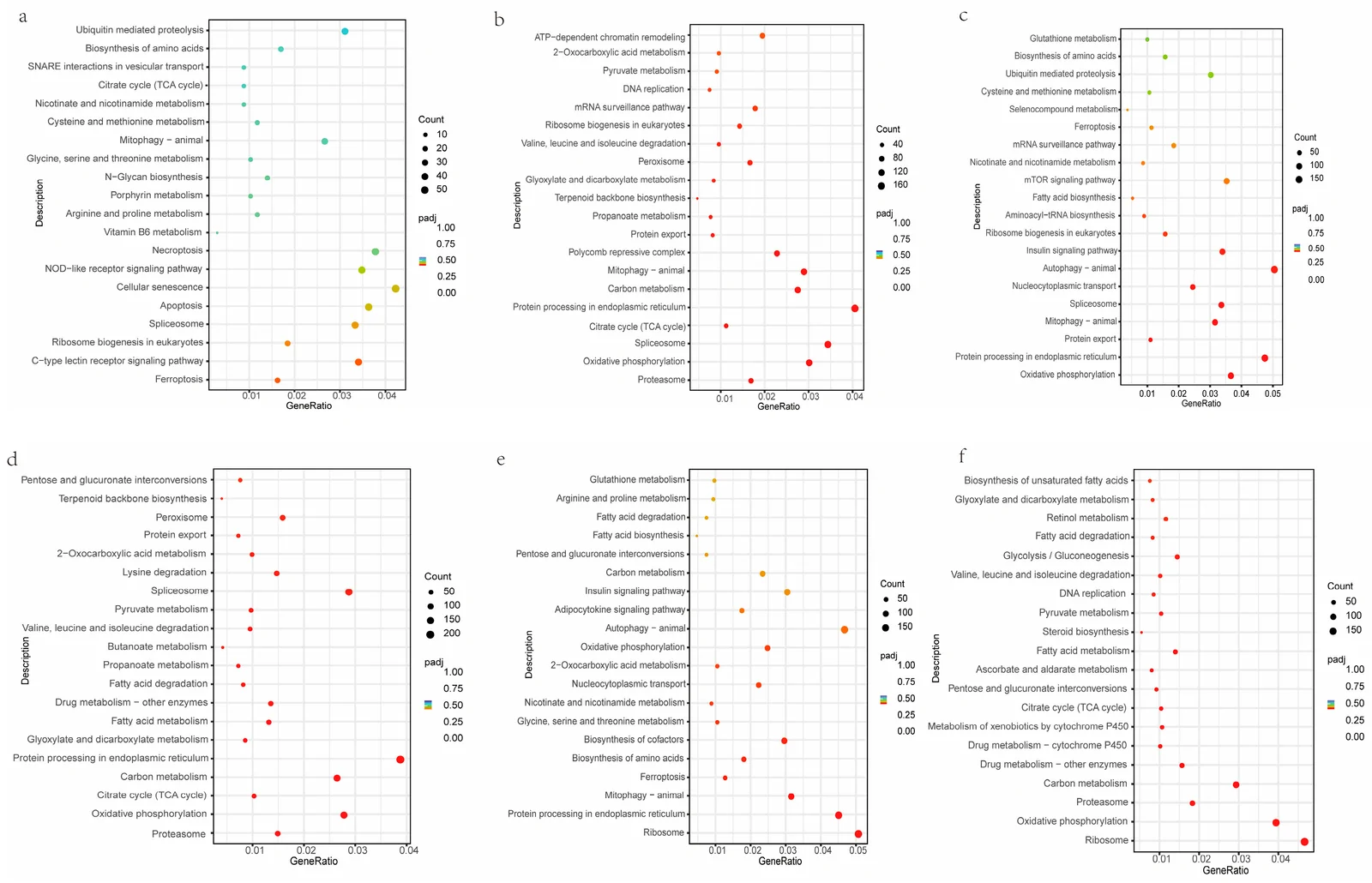
KEGG classification of differentially expressed genes. Notes: (**a**) LLF vs. LLM; (**b**) LLF vs. LLA; (**c**) LLF vs. LLN; (**d**) LLM vs. LLA; (**e**) LLM vs. LLN; (**f**) LLA vs. LLN. Bubble size represents the number of enriched genes, and bubble color corresponds to adjusted *p*-value (padj).

The top 20 most significantly enriched KEGG pathways (*p* < 0.05) were visualized using a bubble plot (Figure 4). Differentially expressed genes (DEGs) in the LLF vs. LLM comparison (Figure 3a) were significantly enriched in Ferroptosis, C-type lectin receptor signaling pathway, and Ribosome biogenesis in eukaryotes. In the LLF vs. LLA group (Figure 3b), DEGs were predominantly enriched in Proteasome, Oxidative phosphorylation, and Spliceosome pathways. For the LLF vs. LLN comparison (Figure 3c), DEGs were significantly associated with Oxidative phosphorylation, Protein processing in endoplasmic reticulum, and Protein export. In the LLM vs. LLA comparison (Figure 3d), key enriched pathways included Proteasome, Oxidative phosphorylation, and the Citrate cycle (TCA cycle). DEGs in the LLM vs. LLN group (Figure 3e) were significantly enriched in Ribosome, Protein processing in endoplasmic reticulum, and Mitophagy—animal pathways. Finally, in the LLA vs. LLN comparison (Figure 3f), DEGs were notably enriched in Ribosome, Oxidative phosphorylation, and Proteasome pathways.

#### 3.1.5. RT-qPCR Validation of Selected Differentially Expressed Genes

**Figure 5:**
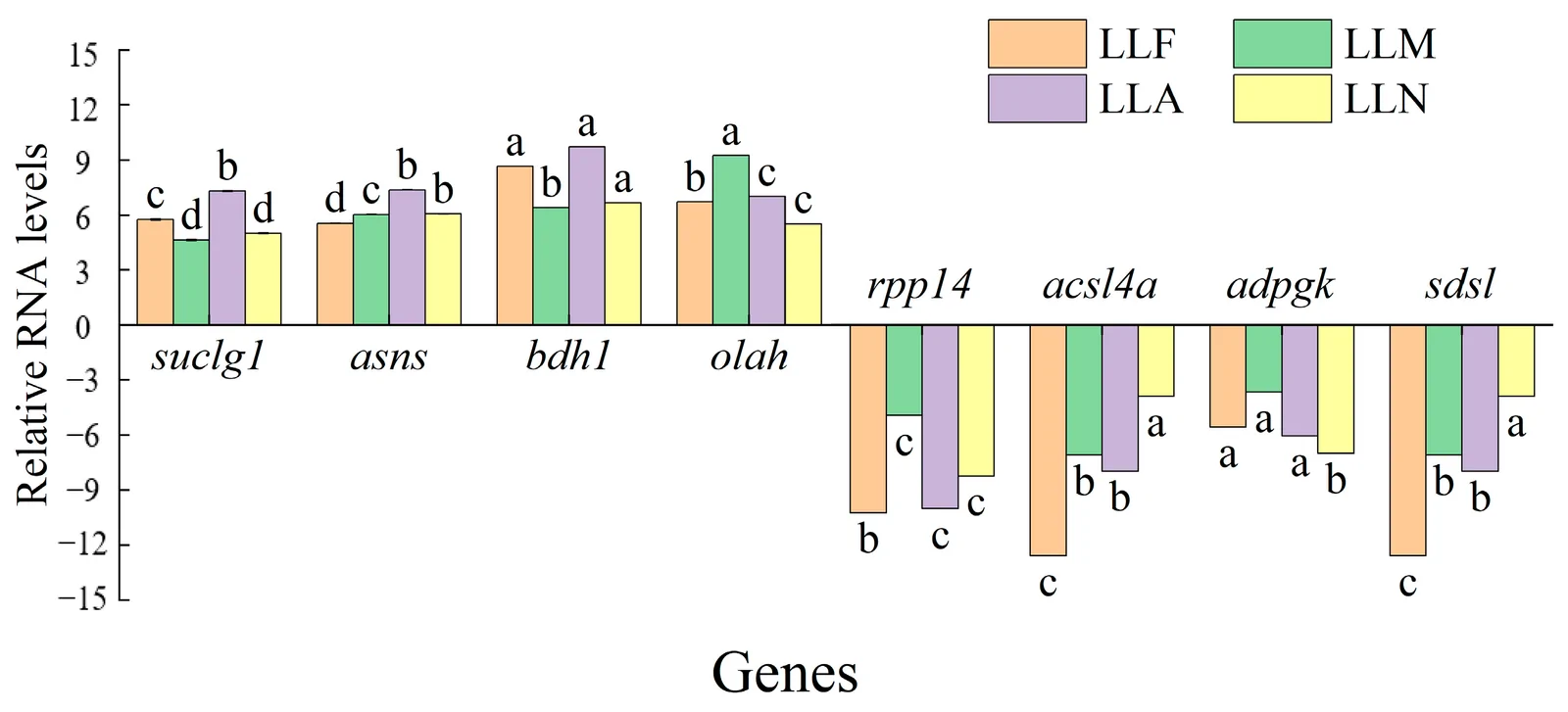
RT-qPCR validation of selected DEGs. Notes: Relative RNA levels (mean ± SD, *n* = 3 per group). RNA levels are normalized to the geometric mean of the reference gene β-actin, with the LLA group set as the calibrator (value = 1.0) for each gene examined. Statistically significant differences between groups (*p* < 0.05) are denoted by different lowercase letters (a, b, c, d).

To assess the reliability of the RNA-seq data, we performed RT-qPCR on a subset of eight DEGs that exhibited substantial fold changes among the four seasonal groups. The RT-qPCR results showed expression trends consistent with the RNA-seq FPKM values across all four groups (LLF, LLM, LLA, and LLN) (Figure 5). Consistent with the transcriptomic results, *suclg*1, *asns*, *bdh*1, and *olah* were consistently upregulated across all four seasonal groups, whereas *rpp*14, *acsl4a*, *adpgk*, and *sdsl* were consistently downregulated, confirming the concordance between the RT-qPCR and RNA-seq expression trends.

### 3.2. Metabolomic Analysis

#### 3.2.1. Analysis of Differential Metabolites

**Figure 6:**
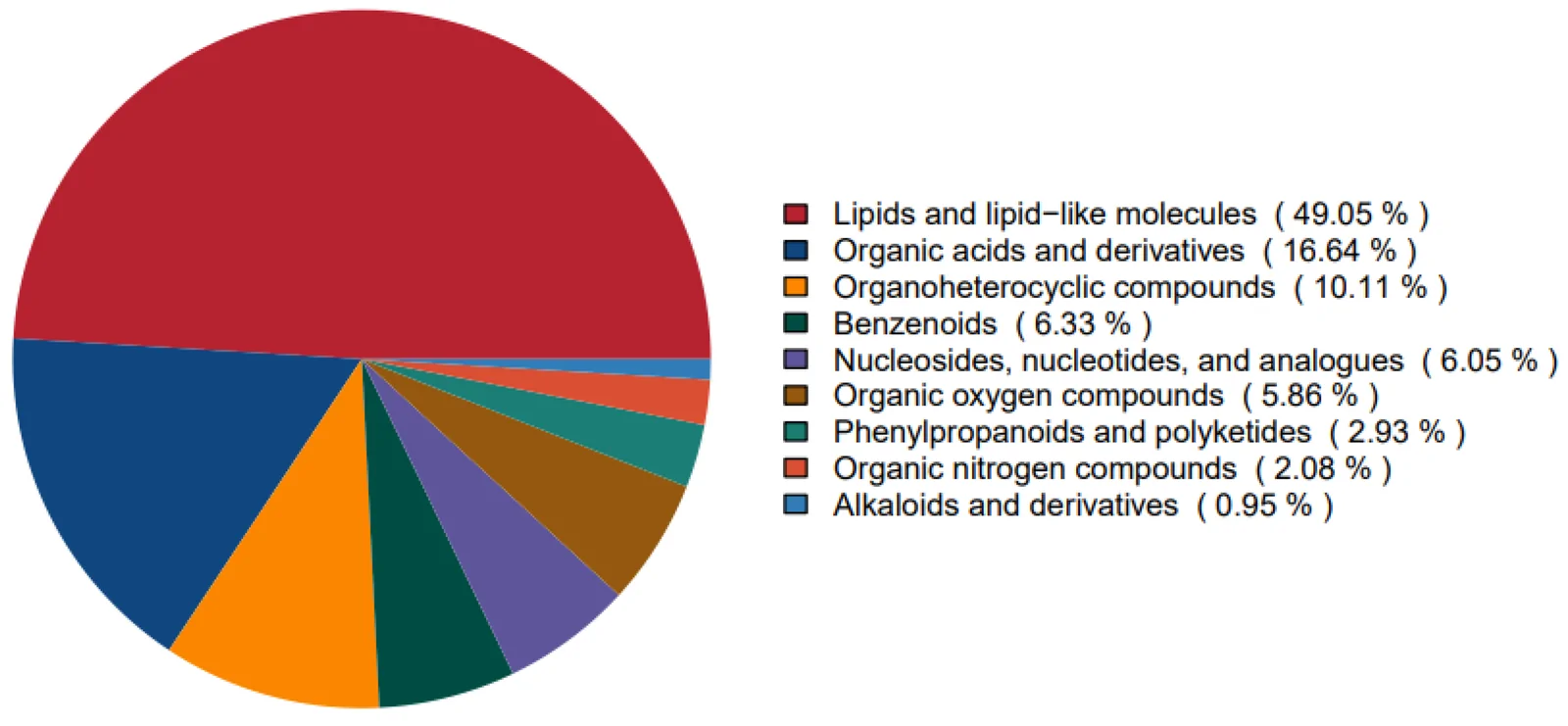
Pie chart of metabolite classification.

Metabolite annotation was conducted, identifying a total of nine metabolite classes (Figure 6). A total of 1058 metabolites were annotated. Lipids and lipid-like molecules represented the largest proportion, comprising 519 metabolites (49.05%), indicating a significant enrichment of lipid species. This was followed by organic acids and their derivatives, which accounted for 176 metabolites (16.64%), suggesting an active involvement in energy metabolism pathways. A notable proportion of organoheterocyclic compounds was also observed, totaling 107 metabolites (10.11%), potentially reflecting the presence of secondary metabolites. In contrast, organic nitrogen compounds (22 metabolites, 2.08%) and alkaloids and their derivatives (10 metabolites, 0.95%) were present in relatively low abundances, indicating limited accumulation of nitrogen-containing and alkaloid-based secondary metabolites under the tested conditions.

#### 3.2.2. PCA of Metabolic Profiles

**Figure 7:**
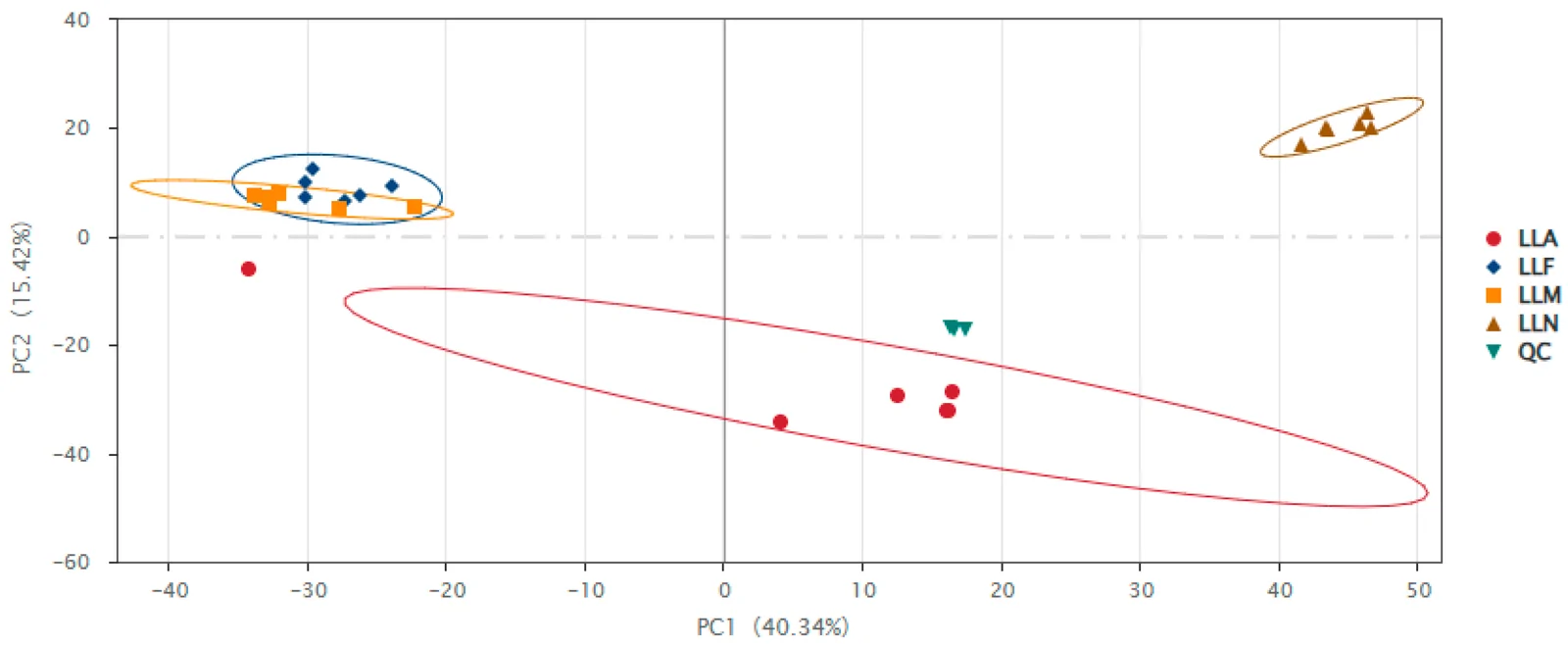
Principal component analysis (PCA) of total samples.

Principal component analysis (PCA) revealed that the quality control (QC) samples clustered tightly, indicating high analytical stability and reliable data quality. Clear separation was observed among the four experimental groups: LLF, LLM, LLA, and LLN. The LLF and LLM groups were situated in relatively close proximity, suggesting detectable yet modest metabolic differences between them. In contrast, the LLA group exhibited a pronounced displacement along the PC1 axis, reflecting a substantial divergence in metabolic composition compared to the other groups. Notably, the LLN group formed a distinct cluster along both principal components (PC1 and PC2), underscoring its unique metabolic profile (Figure 7). Within each seasonal group, the six biological replicates clustered with reasonable coherence along both principal components, indicating that seasonal variation constituted the dominant source of metabolomic variance in this dataset.

#### 3.2.3. Analysis of Differentially Abundant Metabolites

**Figure 8:**
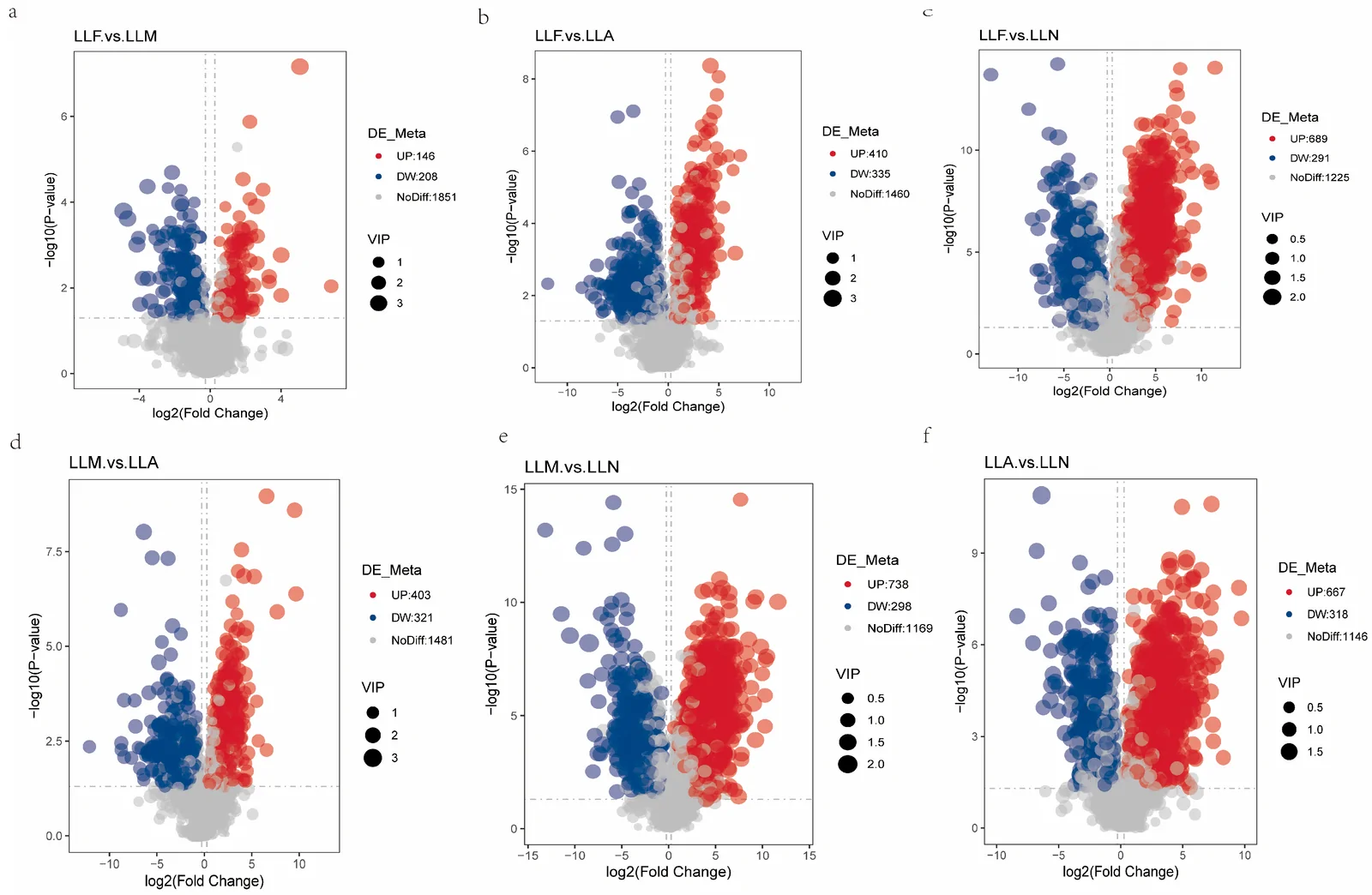
Volcano plot of differential metabolites. Notes: (**a**) LLF vs. LLM; (**b**) LLF vs. LLA; (**c**) LLF vs. LLN; (**d**) LLM vs. LLA; (**e**) LLM vs. LLN; (**f**) LLA vs. LLN. Vertical dashed lines represent |log_2_ (Fold Change)|, and the horizontal dashed line represents FDR = 0.05 (−log_10_(*p*-value)).

In most comparisons, the number of upregulated metabolites exceeded that of downregulated metabolites, with the sole exception of the LLF vs. LLM group (Figure 8a), where downregulated metabolites were more prevalent. The highest numbers of differentially accumulated metabolites were observed in comparisons involving the LLN group: 1036 in the LLM vs. LLN comparison (Figure 8e), 985 in the LLA vs. LLN comparison (Figure 8f), and 980 in the LLF vs. LLN comparison (Figure 8c), with upregulation dominating in all three instances (71.2%, 67.7%, and 70.3%, respectively). In contrast, the fewest differentially accumulated metabolites were identified in the LLF vs. LLM group comparison (356 metabolites), where downregulation was predominant (58.4%). These results highlight a significant seasonal influence on the metabolome, with the LLN group exhibiting the most extensive metabolic reprogramming—primarily toward upregulation—while the transition between the LLF and LLM groups demonstrates a more subdued and predominantly downregulated response.

#### 3.2.4. KEGG Pathway Enrichment of Differentially Abundant Metabolites

**Figure 9:**
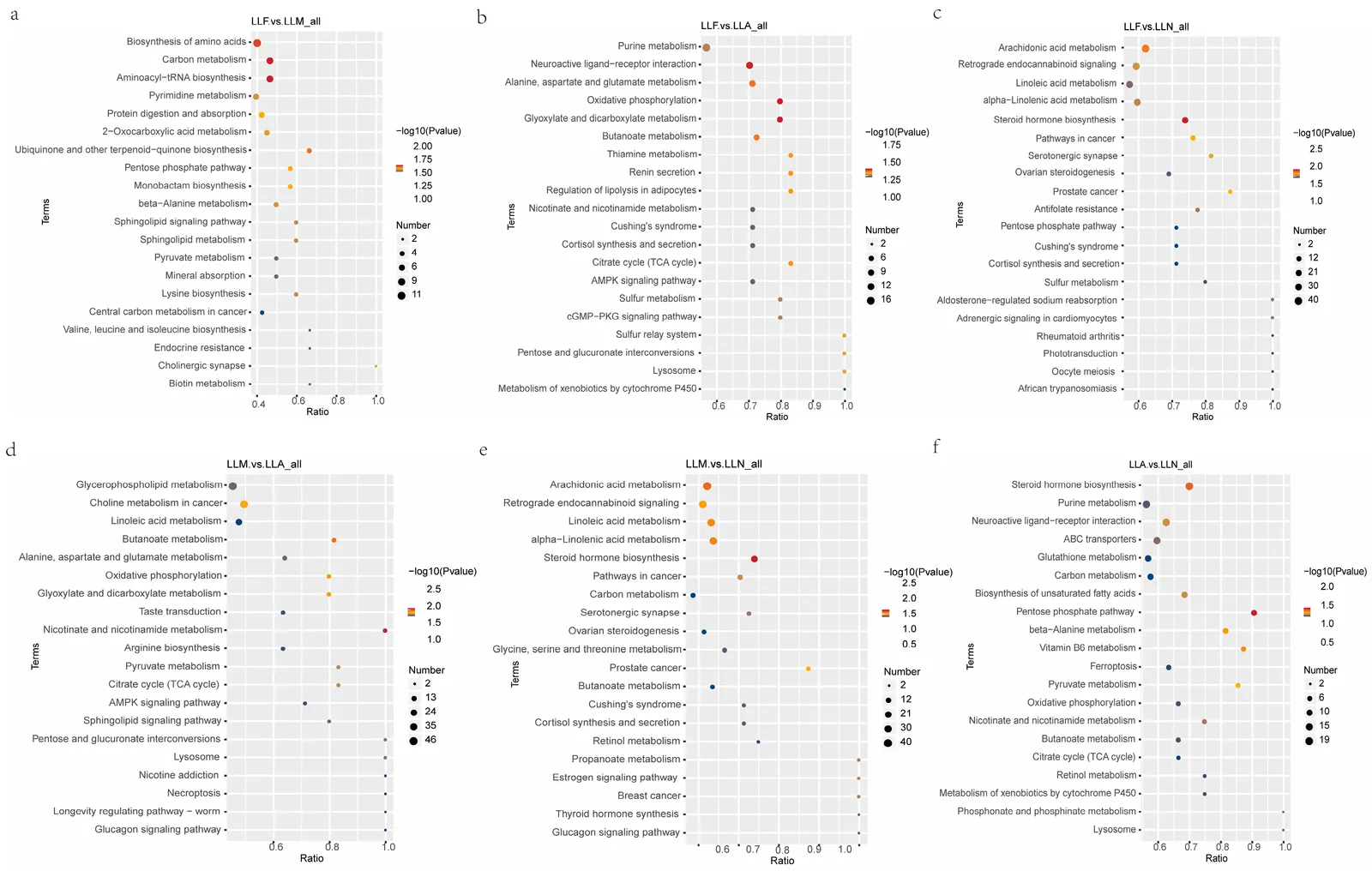
KEGG Pathway Enrichment Bubble Plot of Differential Metabolites. Notes: (**a**) LLF vs. LLM; (**b**) LLF vs. LLA; (**c**) LLF vs. LLN; (**d**) LLM vs. LLA; (**e**) LLM vs. LLN; (**f**) LLA vs. LLN.

KEGG enrichment analysis of differentially accumulated metabolites across seasonal comparisons revealed that the LLN group exhibited the most extensive pathway alterations, indicating the strongest metabolic divergence from other seasons. The comparison between the LLF and LLM groups (Figure 9a) showed the fewest changes, primarily involving amino acid metabolism, such as Aminoacyl-tRNA biosynthesis and Carbon metabolism. In contrast, the comparison between the LLF and LLA groups (Figure 9b) enriched energy metabolism and signaling pathways, notably Oxidative phosphorylation and Neuroactive ligand-receptor interaction. All comparisons involving the LLN group (including LLF vs. LLN group (Figure 9c), LLM vs. LLN group (Figure 9e), and LLA vs. LLN group (Figure 9f)) convergently enriched lipid metabolism pathways, including Steroid hormone biosynthesis and Arachidonic acid metabolism. The comparisons of the LLM vs. LLN group and LLA vs. LLN group additionally implicated alpha-Linolenic acid and Linoleic acid metabolism. Among these, the pathways of interest—Arachidonic acid metabolism and alpha-Linolenic acid metabolism—were consistently enriched in all comparisons involving the LLN group. Furthermore, Oxidative phosphorylation was commonly enriched in both metabolomic (LLF vs. LLA group) and transcriptomic datasets, as shown in previous KEGG results of differentially expressed genes (DEGs). Collectively, these results demonstrate that autumn (LLN) drives a pronounced lipid-centric metabolic reprogramming, whereas transitions from winter to spring and spring to summer are characterized by shifts in amino acid and energy metabolism, respectively.

### 3.3. Integrated Transcriptomic and Metabolomic Analysis

**Figure 10:**
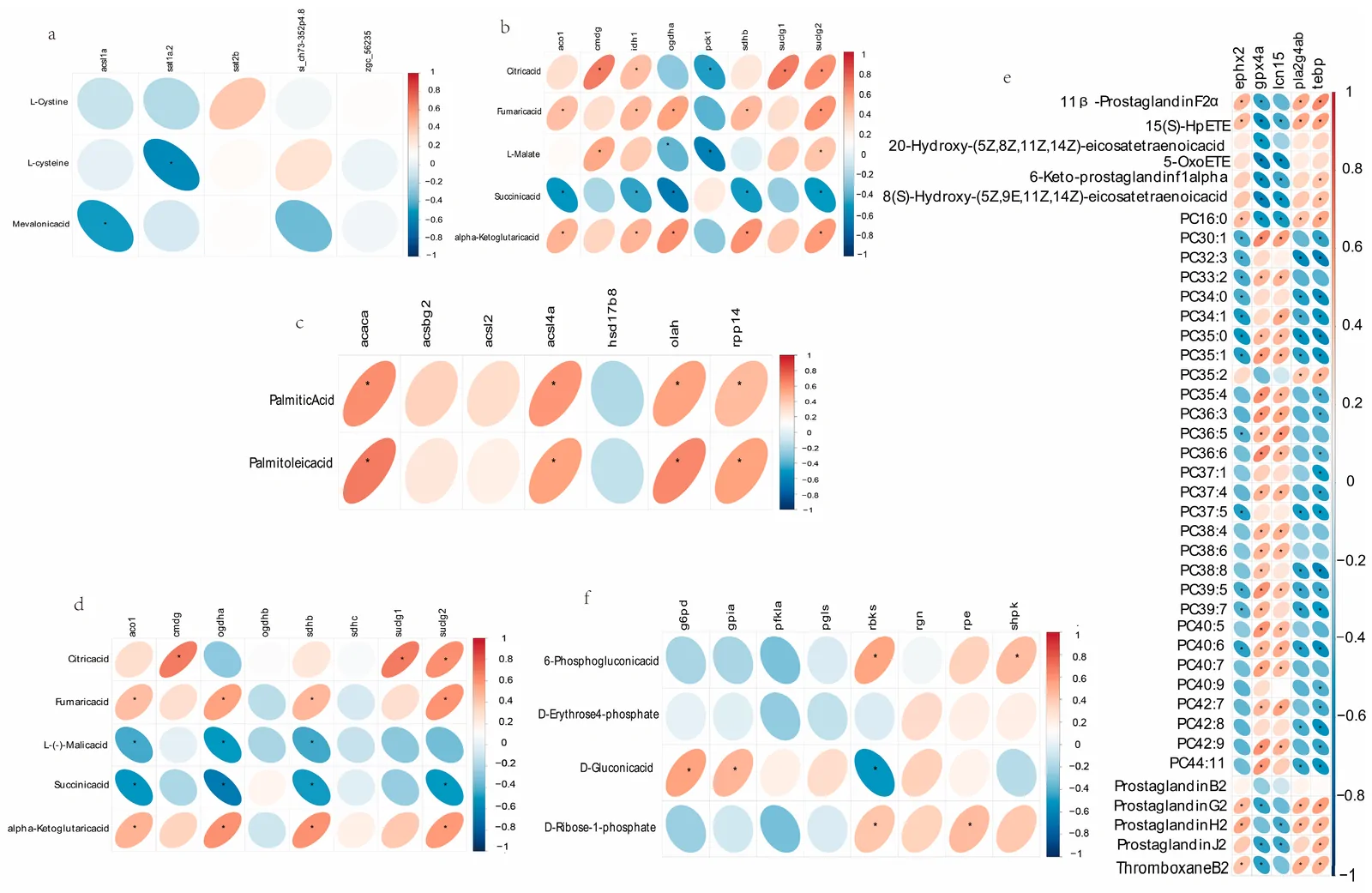
Pearson correlation analysis of seasonal metabolomic and transcriptomic datasets. Notes: (**a**) LLF vs. LLM; (**b**) LLF vs. LLA; (**c**) LLF vs. LLN; (**d**) LLM vs. LLA; (**e**) LLM vs. LLN; (**f**) LLA vs. LLN. Colors indicate the correlation coefficient (r), ranging from –1 (blue) to +1 (red); only gene–metabolite pairs with *p* < 0.05 are shown. An asterisk (*) indicates a statistically significant difference at *p* < 0.05.

To explore the regulatory networks underlying seasonal adaptation, we conducted Pearson correlation analysis between differentially expressed genes and differential metabolites across selected pathways (Figure 10). The correlation heatmaps revealed complex co-expression relationships characterized by pathway-specific patterns. The Ferroptosis pathway was predominant during the transition from the LLF to the LLM group (Figure 10a), In the transition from the LLF to the LLA group (Figure 10b), the TCA cycle emerged as the most seasonally responsive, with multiple genes positively correlated with citric acid, fumaric acid, L-malaate, succinic acid, and alpha-ketoglutaric acid (Figure 10b) Palmitic acid, and Palmitolei acid displayed strong positive correlations (r > 0.6) with multiple variables, further supporting the activation of energy metabolism during the summer transition. In the comparison between the LLF and LLN groups (Figure 10c), the TCA cycle emerged as the most seasonally responsive, with multiple genes positively correlated with citric acid, fumaric acid, L-malic acid, succinic acid, and alpha-ketoglutaric acid, suggesting a concerted activation of energy metabolism under warming conditions. During the transition from the LLM to the LLA group (Figure 10d), fumaric acid, L-malic acid, and succinic acid displayed strong positive correlations (r > 0.6) with multiple variables, further supporting the activation of energy metabolism during the summer transition. For the transition from the LLM to the LLN group (Figure 10e), the Arachidonic Acid Metabolism pathway exhibited strong positive correlations between all detected metabolites (e.g., 11β-prostaglandin F2α, 15(S)-HpETE, prostaglandins, thromboxane B2) and PC-class phospholipids, indicating a coordinated enhancement of phospholipid-derived signaling molecules. In the comparison between LLA and LLN (Figure 10f), in the pentose phosphate pathway panel, *rbks* exhibited a strong positive correlation with 6-phosphogluconic acid but a strong negative correlation with D-gluconic acid, while *g6pd* and *gpia* were both positively correlated with D-gluconic acid. D-ribose-1-phosphate showed positive correlations with both *rbks* and *rpe*, suggesting coordinated regulation of ribose metabolism, whereas D-erythrose-4-phosphate displayed no statistically significant correlation with any of the annotated genes in this pathway. This indicates a shift in the utilization of nitrogen-related metabolites. Collectively, these network analyses identify season-specific regulatory hubs—Ferroptosis, TCA cycle, Fatty acid biosynthesis, Arachidonic acid metabolism, and Pentose phosphate pathway—each of which plays a dominant role during distinct seasonal transitions.

## 4. Discussion

The integrated transcriptomic and metabolomic analyses presented in this study elucidate the molecular adaptive strategies of large-sized triploid rainbow trout (*Oncorhynchus mykiss*) in response to seasonal environmental variation, demonstrating that these fish accommodate seasonal thermal fluctuations through the dynamic and coordinated regulation of energy, amino acid, and lipid metabolism networks—findings that collectively advance our mechanistic understanding of seasonal acclimatization in cold-water fish species. It should be acknowledged, however, that because sampling was necessarily conducted under natural field conditions, water temperature was the only environmental variable continuously monitored across seasons; other seasonally covarying parameters—including photoperiod, dissolved oxygen concentration, and water-column nutrient availability—were not concurrently recorded. As natural seasonal transitions in temperate reservoirs entail coordinated shifts across multiple abiotic variables, the potential contribution of these factors to the observed hepatic transcriptomic and metabolomic changes cannot be fully excluded. The molecular responses reported herein should therefore be interpreted as reflecting an integrated, ecologically realistic seasonal acclimatization response rather than as effects attributable exclusively to water temperature, and future studies employing controlled factorial designs are warranted to delineate the relative contributions of individual environmental drivers.

### 4.1. Biological Significance of the Seasonal Adaptation Mechanism in Triploid Rainbow Trout

The comparison between the warm season (August) and the cold season (February) revealed the number of differentially expressed genes (DEGs; 13,359) and differentially expressed metabolites (DEMs; 745). DEGs were significantly enriched in Integrated network analyses revealed that the TCA cycle, fatty acid biosynthesis, arachidonic acid metabolism, pentose phosphate pathway, and ferroptosis function as seasonally dominant regulatory hubs, with pathway-specific gene–metabolite correlations supporting a coordinated metabolic reprogramming underlying seasonal adaptation in triploid rainbow trout. The coordinated increase in arachidonic-acid-derived eicosanoids (prostaglandins, thromboxane B2) alongside PC-class phospholipids during the LLM-to-LLN transition may reflect membrane phospholipid remodeling and eicosanoid-mediated immune/inflammatory signaling as water temperature declines into autumn, a response broadly consistent with cold-induced membrane fluidity adjustment and stress–immune modulation described in other teleosts. Similarly, the divergent correlations within the pentose phosphate pathway—positive for 6-phosphogluconate but negative for D-gluconic acid with *rbks*—may indicate a redirection of glucose-6-phosphate flux toward NADPH and ribose-phosphate production rather than toward gluconate accumulation, consistent with heightened oxidative and biosynthetic demand during the LLA-to-LLN transition. This suggests that triploid rainbow trout respond to seasonal temperature by coordinating enhancements in energy metabolism and antioxidant defense. This finding aligns with the work of Mir et al. [25], which reported season-dependent changes in glucose, triglyceride and GK levels in rainbow trout. It should be noted, however, that their study was performed on diploid fish rather than triploids; hence, these observations represent general seasonal metabolic characteristics of rainbow trout and may not be triploid-specific. Additionally, temperature-dependent regulation of GK expression and glucose metabolism in rainbow trout has been demonstrated under varying thermal conditions [26].

In the comparison between February and November, differentially expressed genes were significantly enriched in anabolic pathways such as ribosome biogenesis and protein processing, while metabolites accumulated at the level of amino acids (e.g., methionine, phenylalanine) and nucleotide metabolites. This observation suggests that during the cold season, triploid rainbow trout prioritize energy allocation towards basal maintenance and protein turnover rather than rapid growth [27]. A similar seasonal metabolic shift was noted in gilthead sea bream, where lipid accumulation occurred from September to February (cold adaptation), and carbohydrate metabolism dominated from February to July (warm period) [28]. In contrast, Feidantsis et al. previously documented a comparable seasonal metabolic pattern, indicating that lipid oxidation is induced during cold acclimation, while warm acclimation triggers metabolic activation and carbohydrate oxidation [29]. This discrepancy—lipid accumulation versus lipid oxidation during cold acclimation—suggests that the direction of lipid metabolic remodeling under seasonal thermal change may be species- or context-dependent, rather than following a universal pattern across teleosts. These metabolic adjustments underscore the adaptive responses of fish to seasonal temperature fluctuations and provide insights into the mechanisms underlying thermal tolerance and energy utilization.

### 4.2. Comparison of Temperature Adaptability Between Triploid and Diploid Rainbow Trout

Previous controlled experiments comparing triploid and diploid rainbow trout have yielded valuable, if not entirely consistent, insights into the thermal biology of triploids. In one such study, triploid rainbow trout displayed an earlier onset of cardiac arrhythmia when subjected to high-temperature stress. All triploid specimens experienced arrhythmia at 22 °C, whereas 30% of diploid specimens sustained normal heart rhythms. This observation implies that triploid trout may exhibit lower tolerance to elevated temperatures, possibly due to compromised oxygen delivery within their cardiovascular systems [30]. Our finding of coordinated upregulation of energy metabolism and antioxidant defense pathways during the warm season is consistent with this reduced thermal margin, suggesting a compensatory molecular response to the cardiovascular limitations reported in diploid-triploid comparisons. However, a follow-up study by the same research team observed no notable differences in blood oxygen affinity between triploid and diploid rainbow trout across different temperatures and CO_2_ partial pressures. This suggests that the hypoxia tolerance of triploids is not constrained by the blood’s capacity to transport oxygen [31]. Triploid rainbow trout generally demonstrate enhanced growth rates as a result of their sterility. Nonetheless, the issue of whether triploid individuals have inferior thermal tolerance compared to diploids remains a topic of discussion. Under prolonged high-temperature stress, all-female triploid rainbow trout exhibited significantly [32] lower survival rates and lost their usual growth advantage in comparison to diploids. Mechanistic investigations indicate that triploids have reduced erythrocyte volumes without a corresponding increase in erythrocyte count, resulting in a restricted oxygen transport capacity. Therefore, raising triploid rainbow trout during the high-temperature summer months presents increased risks and requires careful management strategies [33]. The existing literature on triploid thermal tolerance thus presents a mixed and still-evolving picture, and caution is warranted in drawing overly generalized conclusions about ploidy-specific differences in thermal performance. While these earlier diploid-triploid comparative studies were based on controlled experimental heat challenges and focused primarily on cardiovascular and hematological parameters, the present study extends this understanding by identifying the underlying hepatic transcriptomic and metabolomic responses of triploid rainbow trout under natural seasonal temperature variation. Our results align with the reduced thermal tolerance reported in these earlier studies, while further revealing candidate molecular hubs—such as the carnosine–histidine module and the pentose phosphate pathway—that were not previously characterized, thereby providing a more detailed mechanistic basis for the compensatory strategies triploid trout may employ under warm-season conditions.

This study offers a novel perspective on metabolic regulation. In comparing the August (LLA) and November (LLN) groups, triploid rainbow trout exhibited a significant activation of the pentose phosphate pathway, as evidenced by the positive correlation of genes such as *g*6*pdh*, *6pgdh*, and *taldo* with D-ribose-1-phosphate. Additionally, the β-alanine metabolism pathway was activated, marked by the accumulation of carnosine and spermine. The activation of these antioxidant defense systems may partially mitigate the physiological limitations of the cardiovascular system in triploid rainbow trout. This finding is consistent with the research conducted by Han et al., which indicated no significant differences in plasma cortisol, glucose, or liver heat shock protein (*Hsp*70) levels between triploid and diploid rainbow trout following transport stress. Similar stress resilience in triploid rainbow trout has also been reported under acute hypoxic conditions in a study conducted on the same commercial stock, further supporting the notion that triploids possess effective acute stress response capabilities [34]. Therefore, triploid rainbow trout may enhance protective metabolic mechanisms, such as the accumulation of antioxidant metabolites, to compensate for their lower thermal tolerance. Similar compensatory metabolic responses have been documented in other studies focusing on stress adaptation in triploid fish [35]. Against this background, the present study extends the mechanistic understanding of seasonal acclimatization in triploid rainbow trout by characterizing hepatic transcriptomic and metabolomic responses under natural field-based temperature variation—an ecologically realistic context not captured by controlled acute heat-challenge paradigms. As emphasized above, however, the absence of concurrent diploid controls in the present study precludes definitive attribution of these responses to triploid-specific physiology, and this mechanistic distinction awaits clarification through appropriately designed comparative experiments.

### 4.3. Seasonal Variation in Immune- and Stress-Related Pathway Regulation

This research additionally uncovered notable seasonal variation in the regulation of immune- and stress-related pathways. Between February and May (LLF vs. LLM), differentially expressed genes were significantly enriched in C-type lectin receptor signaling, a pathway broadly implicated in innate immune recognition in teleosts. Between May and November (LLM vs. LLN), significant enrichment was observed in arachidonic- and linoleic-acid metabolism, accompanied by broad positive correlations between prostaglandin-class metabolites and phospholipids (Section 3.3), consistent with a shift toward eicosanoid-mediated immune/inflammatory signaling as temperature declined into autumn. In the present dataset, genes linked to protein synthesis and energy production were significantly upregulated in the liver during the warm season; a comparable pattern of enhanced protein-turnover-related gene expression in skeletal muscle has been reported in triploid salmonids under size-related physiological stress [36], suggesting that the liver-centered response identified here may form part of a broader, multi-organ strategy that warrants direct investigation in future work. Under prolonged warm-season conditions, the gene expression patterns observed in this study are consistent with a strategy that prioritizes survival-related maintenance functions over growth, underscoring the potential value of enhanced management during high-temperature periods and offering candidate molecular targets for breeding programs aimed at improving thermotolerance. This outcome underscores the necessity for enhanced management techniques during high-temperature aquaculture, offers potential molecular targets for breeding for thermotolerance, and enriches our theoretical perspective on triploid adaptation by highlighting “adaptive compensation” instead of “inherent deficiency” [37].

This investigation introduces one of the first integrative multi-omics assessments to characterize the molecular regulatory framework underlying seasonal acclimatization in large triploid rainbow trout. The findings reveal that, during the warm season, energy metabolism and antioxidant defense pathways were concurrently upregulated, suggesting a coordinated, synergistic strategy for physiological adaptation. Notably, the carnosine–histidine module and the pentose phosphate pathway emerged as candidate regulatory hubs potentially associated with warm-season physiological adjustment. Although triploid and diploid rainbow trout were not directly compared within this study, these findings provide a molecular-level reference that may help contextualize the differences in seasonal conditions previously reported between triploid and diploid rainbow trout in the literature. Overall, this research contributes to a deeper theoretical understanding of how cold-water commercial fish species adapt to their environment and lays the groundwork for breeding strains that are resilient to environmental fluctuations and for developing targeted aquaculture management strategies.

### 4.4. Limitations

Several limitations of this study warrant acknowledgement. First, the per-season sample size was relatively small (*n* = 6 fish), which may have constrained statistical power in detecting subtler transcriptomic and metabolomic changes and limited the broader generalizability of the findings. Second, all specimens were sampled from a single reservoir (Longyangxia Reservoir, Qinghai Province) within a single cohort, such that site- or population-specific environmental and genetic factors cannot be excluded as contributors to the observed seasonal patterns; cross-validation across multiple geographically distinct sites and independent cohorts is therefore warranted. Third, the sampled fish spanned a relatively wide body-mass range (2–3 kg), which may have introduced inter-individual variability not directly attributable to seasonal effects. Fourth, because sampling was conducted under natural field conditions, only water temperature was continuously recorded; other seasonally covarying environmental factors, such as photoperiod, dissolved oxygen, and water-column nutrient status, were not monitored and may have contributed to the observed molecular changes. Fifth, fish were fasted for 24 h prior to sampling to standardize feeding status across groups; however, natural feeding intensity differs substantially across seasons, and this controlled fasting period may itself introduce a degree of systematic bias into the hepatic metabolic background relative to free-feeding conditions in the wild. Finally, as with most untargeted metabolomics studies, a proportion of the detected metabolite features could not be unambiguously annotated against reference databases, which may have limited the completeness of pathway-level interpretation. Future studies incorporating larger sample sizes, multiple rearing sites, parallel diploid control groups, standardized feeding and photoperiod protocols, narrower body-size ranges, and targeted metabolomic validation of key candidate antioxidants such as carnosine and spermine would help to further validate and refine the molecular mechanisms identified here.

## 5. Conclusions

This study employed integrated transcriptomic and metabolomic analyses to characterize the seasonal molecular adaptation of large-sized triploid rainbow trout (*Oncorhynchus mykiss*). Fish prioritized energy metabolism and antioxidative defense during the warm season, whereas cold months were characterized by pathways supporting basic maintenance functions, with pronounced lipid metabolic remodeling in autumn. Five season-specific regulatory hubs were identified—ferroptosis, the TCA cycle, fatty acid biosynthesis, arachidonic acid metabolism, and the pentose phosphate pathway—with autumn showing pronounced lipid metabolic reprogramming. These findings advance understanding of environmental adaptation in cold-water fish and provide molecular targets for breeding stress-resistant triploid rainbow trout and precision aquaculture management.

## Figures and Tables

**Table 1:** Primer information for qRT-PCR.

Name of Primers	Sequence of Primers	Expression Trend
*actb*	CTACCTGATGAAGATCCTGACGG	
	CAGCTTCTCCTTGATGTCTCGTA	
*suclg1*	TGGAGAGCCATTCAAGCGTT	Up
	ACTCCCCACCATTGCTTCTG	
*asns*	TCAGCGACGGAGGAAAG	Up
	AAGTAGGCAGGGACTGTGAT	
*bdh1*	GGGAAGGCTCCTACTCG	Up
	TTGATGGGCACCACTCT	
*olah*	ACATTGCCAACCTCATCATCG	Up
	TTGAGCAGGTCCTTGTCCTTG	
*rpp14*	AGAATGTCAGCCAGCCTTGT	Down
	TCTCAGACTCATCCCCTCAGT	
*acsl4a*	AGGACCTGGACCTGGAGATGTTG	Down
	CGCACAGAAGCACAGAGAAGAG	
*adpgk*	CTCGTGCCTACTACATCTGCT	Down
	ACCGTAAAGGAAAACCCCAG	
*sdsl*	TTCTCCACCACAARGAYATYGG	Down
	CTCAGGGTGTGAARAAYCARAA	

**Table 2:** Transcriptome sequencing data.

Sample	Raw_reads	Raw_bases	Clean Reads	Clean_bases	GC (%)	Q20 (%)	Q30 (%)
LLF1	47,417,772	7.11 G	45,146,548	6.77 G	49.75	98.39	95.82
LLF2	50,276,620	7.54 G	47,787,098	7.17 G	49.82	98.38	95.76
LLF3	46,180,172	6.93 G	44,734,922	6.71 G	47.17	98.46	95.96
LLF4	45,175,014	6.78 G	43,212,412	6.48 G	49.01	98.32	95.62
LLF5	47,971,196	7.2 G	46,145,140	6.92 G	49.44	98.47	96.03
LLF6	46,482,392	6.97 G	44,385,872	6.66 G	49.11	98.31	95.67
LLM1	45,312,820	6.8 G	43,360,960	6.5 G	49.6	98.37	95.7
LLM2	50,021,454	7.5 G	47,726,904	7.16 G	49.01	98.4	95.84
LLM3	42,351,308	6.35 G	40,568,632	6.09 G	50.02	98.26	95.5
LLM4	49,470,844	7.42 G	47,093,810	7.06 G	49.38	98.37	95.8
LLM5	47,722,214	7.16 G	45,663,190	6.85 G	49.35	98.61	96.32
LLM6	49,042,842	7.36 G	46,083,226	6.91 G	49.07	98.41	95.85
LLA1	41,190,394	6.18 G	39,095,958	5.86 G	47.69	98.75	96.81
LLA2	40,917,924	6.14 G	39,047,408	5.86 G	47.6	98.75	96.8
LLA3	47,857,156	7.18 G	45,846,658	6.88 G	48.11	98.72	96.73
LLA4	39,310,064	5.9 G	37,495,668	5.62 G	46.38	98.73	96.77
LLA5	40,554,994	6.08 G	37,903,368	5.69 G	48.54	98.86	96.99
LLA6	46,145,486	6.92 G	44,275,250	6.64 G	47.78	97.91	94.11
LLN1	44,710,924	6.71 G	43,071,612	6.46 G	50.35	98.98	97.22
LLN2	46,309,544	6.95 G	43,664,416	6.55 G	46.6	98.93	97.23
LLN3	46,485,058	6.97 G	44,048,536	6.61 G	50.87	98.91	97.08
LLN4	48,110,948	7.22 G	45,519,788	6.83 G	49.99	98.9	97.07
LLN5	40,228,500	6.03 G	38,283,386	5.74 G	48.77	98.99	97.2
LLN6	47,894,210	7.18 G	45,433,508	6.82 G	49.54	98.83	96.95

## Data Availability

The raw transcriptome sequencing data have been deposited in the NCBI Sequence Read Archive (SRA) and are accessible under BioProject accession number PRJNA1295884 (https://www.ncbi.nlm.nih.gov/bioproject/PRJNA1295884, accessed on 15 September 2026). The metabolomics dataset generated during the current study is available in the MetaboLights repository (https://www.ebi.ac.uk/metabolights/, accessed on 15 September 2026) under the accession ID MTBLS15014. The complete data can be accessed at https://www.ebi.ac.uk/metabolights/MTBLS15014, accessed on 15 September 2026.

## Abbreviations

The following abbreviations are used in this manuscript: LLF, large-sized triploid rainbow trout collected in late February; LLM, large-sized triploid rainbow trout collected in late May; LLA, large-sized triploid rainbow trout collected in late August; LLN, large-sized triploid rainbow trout collected in late November; DEGs, differentially expressed genes; GO, Gene Ontology; KEGG, Kyoto Encyclopedia of Genes and Genomes; RIN, RNA Integrity Number; FDR, false discovery rate; TCA, tricarboxylic acid.
